# Tubulocystic Renal Cell Carcinoma: An Underrecognized Clinicopathologic Entity

**DOI:** 10.31486/toj.21.0065

**Published:** 2022

**Authors:** Sudeep Khera, Poonam Elhence, Taruna Yadav, Himanshu Pandey

**Affiliations:** ^1^ Department of Pathology, All India Institute of Medical Sciences, Jodhpur, India; ^2^ Department of Diagnostic and Interventional Radiology, All India Institute of Medical Sciences, Jodhpur, India; ^3^ Department of Urology, All India Institute of Medical Sciences, Jodhpur, India

**Keywords:** *Carcinoma–renal cell*, *cyst fluid*, *cysts*

## Abstract

**Background:** Tubulocystic renal cell carcinoma is a lesser-known neoplastic entity compared to other common histologic variants of renal cell carcinoma. The World Health Organization identified tubulocystic renal cell carcinoma as a newly recognized renal tumor in 2016. We report a case of tubulocystic renal cell carcinoma in a young adult.

**Case Report:** A 21-year-old male presented with the chief complaint of a lump on the right side of his abdomen since childhood. Magnetic resonance imaging revealed an enlarged right kidney with multiple large multiloculated cysts with hemorrhagic contents and enhancing peripheral nodular solid components with enhancing septa in some of the cysts, suggesting the possibility of multifocal intracystic papillary renal cell carcinoma. Imaging showed a Bosniak type IV cystic lesion in the right kidney. Right radical nephrectomy was performed. Grossly, the kidney was almost replaced by variable-sized cystic lesions with thick septations filled with serous fluid to gelatinous material. The tubules and cysts were lined by a single layer of flat, hobnail, and cuboidal cells with high-grade nuclear features. No ovarian-type stroma was identified. In places, a papillary component was also identified. Tubulocystic renal cell carcinoma was diagnosed based on microscopy and immunohistochemistry results.

**Conclusion:** Tubulocystic renal cell carcinoma is a rare entity that was previously called Bellini duct carcinoma and low-grade collecting duct carcinoma. Because of the limited number of cases reported, tubulocystic renal cell carcinoma needs to be followed to determine the biologic behavior and metastatic potential of these tumors so that management guidelines for such cases can be developed.

## INTRODUCTION

Tubulocystic renal cell carcinoma is an uncommon and rare variant of renal cell carcinoma that can pose diagnostic difficulties. In 2009, Amin et al^1^ described a series of 31 tumors that they referred to as “tubulocystic carcinoma.”^2^ In 2010, the American Joint Committee on Cancer recognized tubulocystic renal cell carcinoma as a distinct entity, and it was included in the Vancouver Classification of Renal Cancer in 2012. The World Health Organization (WHO) modified the classification of renal cell carcinoma and identified tubulocystic renal cell carcinoma as a newly recognized renal tumor in 2016.^3,4^ The tumor has a predilection for male patients, with a male:female ratio of 7:1, and predominantly involves the left kidney.^5^ No specific risk factors have been identified for this rare tumor. Most patients are asymptomatic on presentation, although hematuria, distention, and abdominal pain are sometimes reported.^6^

Diagnosis of cystic neoplasms of the kidney is challenging, both clinically and radiologically. Histopathology is crucial in establishing the diagnosis. We report the case of a patient who presented with a large multiloculated solid cystic mass in the right kidney.

## CASE REPORT

A 21-year-old male presented with complaints of a right flank mass that had progressively increased in size since childhood and was associated with dull aching pain for the prior 2 years. The patient also complained of constipation and burning micturition. Examination revealed a large nontender lump that was approximately 18 × 12 cm, firm to hard in consistency, and with well-defined borders and side-to-side mobility involving the right hypochondrium, lumbar, and iliac fossa regions. The mass also extended to the epigastric, hypogastric, and umbilical regions. The patient had no history of notable weight loss or loss of appetite. Renal function tests were normal. Bacteriologic examination of the urine did not identify any microorganism growth. Urine samples were sent on 3 consecutive days for malignant cytology examination but were reported as unsatisfactory for evaluation because of the lack of an adequate number of urothelial cells as per the Paris System for Reporting Urinary Cytopathology criteria.^7^

Abdominal ultrasound showed an enlarged right kidney with multiple variable-sized cysts. Hypoechoic heterogeneous contents were seen in 3 to 4 cysts. One to 2 cysts showed multiple septations with a small solid component. Except for the presence of a few small cystic lesions, the left kidney was the expected size.

**Figure 1.**
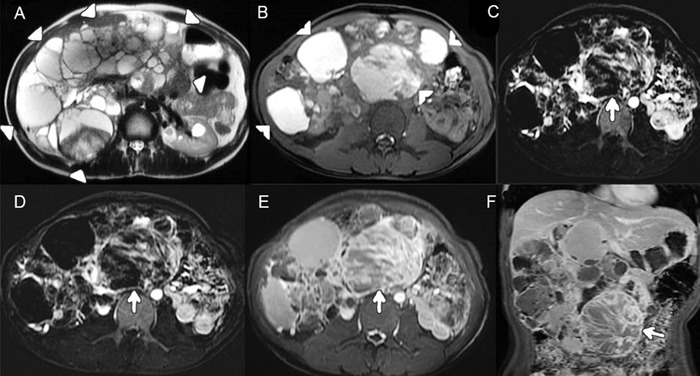
(A) Axial T2-weighted image shows markedly enlarged right kidney (outlined by triangles) studded with multiple cysts, a few containing heterogeneous contents. Some cystic lesions are also seen in the normal sized left kidney. (B) In this axial T1-weighted image, some cysts show T1 hyperintensity suggestive of hemorrhagic/high mucin contents (arrowheads). (C) Corticomedullary phase and (D) nephrographic phase postcontrast (subtracted) axial T1-weighted images reveal progressive enhancement in one of the cysts (arrows) with multiple enhancing septations. (E) Axial 3-minute and (F) coronal 5-minute delayed postcontrast (nonsubtracted) T1-weighted images show further progressive enhancement of the wall of the cystic lesion and septations.

Contrast-enhanced magnetic resonance imaging showed that the right kidney was replaced with innumerable cysts, many of them showing T1 hyperintense signal, and fluid-fluid levels consistent with hemorrhage or high mucinous contents within the cysts. One of the larger cysts and 1 to 2 smaller cysts showed a peripheral enhancing solid component and multiple enhancing internal septations (Figure 1). Four to 5 cystic lesions were characterized as Bosniak category IV cystic renal masses. The renal capsule was intact, with no invasion of adjacent structures. The inferior vena cava was compressed at the right kidney level, accompanied by the formation of multiple right perirenal collaterals. Multifocal cystic renal malignancy in the right kidney was established on imaging with a possibility of intracystic papillary renal cell carcinoma.

**Figure 2.**
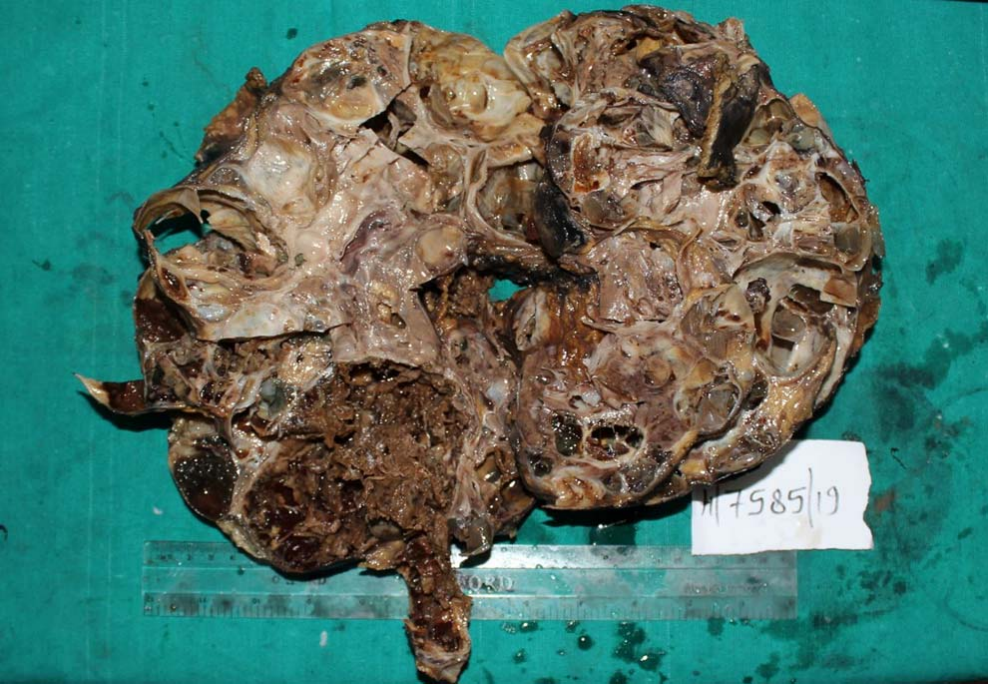
A large multiloculated solid cystic mass entirely replaced the whole of the renal parenchyma.

The patient underwent right radical nephrectomy with a clinical-radiologic diagnosis of malignant cystic neoplasm. A multiloculated solid cystic mass that replaced the entire right kidney was sent for histopathologic evaluation. The surgical specimen measured 37 × 19 × 10 cm and weighed 3,200 g, with the ureter measuring 6 cm in length (Figure 2). The outer surface was lobulated. The brown, fleshy tumor had numerous large and small cysts, ranging in size from 1 cm to 7 cm and filled with clear to hemorrhagic fluids. Three to 4 cysts were also filled with gelatinous fluid. Friable papillary projections were identified in 1 to 2 solid areas, with the largest solid area in the lower pole measuring 9 × 7 × 6 cm. The overlying renal capsule was intact, the pelvicalyceal system was dilated, and the tumor reached the renal pelvis.

**Figure 3.**
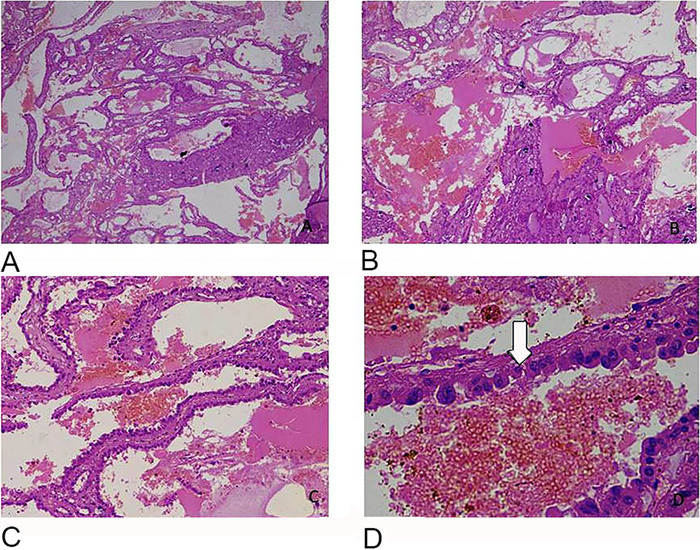
(A, B) Microscopy reveals both cyst structures and small tubules with intervening stroma (A: hematoxylin and eosin [H&E], magnification ×4; B: H&E, magnification ×10). (C, D) Cystic spaces are lined by cuboidal to low columnar eosinophilic epithelial cells with hobnailing (indicated with arrow) and high-grade nuclear features (C: H&E, magnification ×10; D: H&E, magnification ×40).

**Figure 4.**
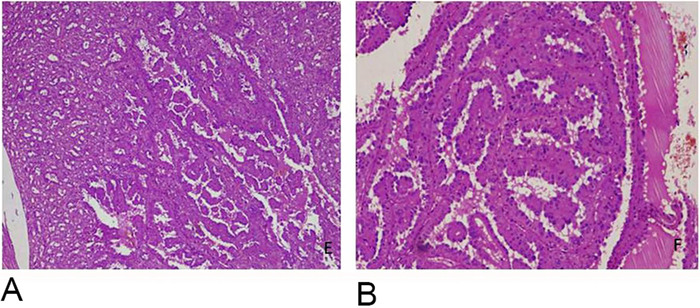
(A) Low-power and (B) high-power magnification show papillary component associated with tubulocystic carcinoma.

The microscopic examination (Figures 3 and 4) revealed a tumor composed of variable-sized tubules, anastomosing papillae, and variable-sized cysts. These cysts were separated by fibrous septa. The tubules and cysts were lined by a single layer of low cuboidal to columnar cells and many hobnail cells. These cells were mild to moderately pleomorphic, having round nuclei, conspicuous nucleoli (International Society of Urological Pathology grade 3), and clear to abundant eosinophilic granular cytoplasm. Areas of calcification and a focal area of ossification were also seen. The solid areas had papillae with fibrovascular cores lined by cuboidal to low columnar cells with moderate to abundant dense, eosinophilic cytoplasm. Focal areas of necrosis were seen. Mitoses were infrequent. No ovarian-type cellular stroma was noted.

The ureter and renal sinus were free of tumor invasion. No lymphovascular or perineural invasions were seen. Eight lymph nodes were resected, and all were free from tumor invasion.

No sarcomatoid or rhabdoid features were identified. The capsule and perinephric fat were free from tumor invasion. Based upon histomorphologic features, a diagnosis of tubulocystic renal cell carcinoma was favored.

**Figure 5.**
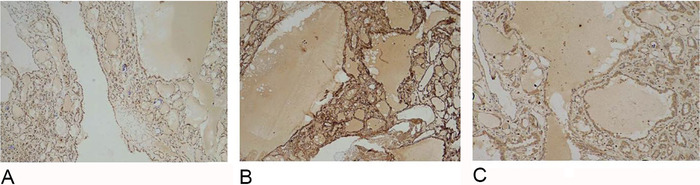
Immunohistochemistry reveals diffuse immunoreactivity (A) for PAX8 (magnification ×4), (B) for vimentin (magnification ×10), and (C) for AMACR (magnification ×10).

Immunohistochemistry panel was performed to confirm the diagnosis and rule out other cystic neoplasms of the kidney with similar morphology. The tumor cells demonstrated immunoreactivity for PAX8, vimentin, and AMACR (Figure 5). The tumor cells were not immunoreactive for CK7 and CD117. No immunoreactivity for estrogen receptors and progesterone receptors was noted in the stroma. Based upon the histomorphologic features and immunohistochemistry profile, a final diagnosis of tubulocystic renal cell carcinoma was made. At 6-month follow-up, the patient had had no recurrence or evidence of metastasis. He did not receive adjuvant therapy. At 1-year follow-up, the patient still had no evidence of recurrence or metastasis.

## DISCUSSION

Tubulocystic renal cell carcinoma is an uncommon tumor; to our knowledge, approximately 70 cases have been reported in the literature.^8^ Masson recognized this tumor as a separate entity based on its histomorphology in 1955, more than 60 years in advance of WHO recognition.^9^ In a series of 13 cases, MacLennan et al reported a detailed description of the tumor, designating it as low-grade mucinous tubulocystic renal cancer, likely of collecting duct origin because of the mucin production.^10^ Low-grade mucinous tubulocystic renal cell carcinomas behave in a less-aggressive fashion than collecting duct carcinomas.

Tubulocystic renal cell carcinoma, included in the 2016 *WHO Classification of Tumours of the Urinary System and Male Genital Organs*,^8^ accounts for <1% of all renal carcinomas and occurs predominantly in males in the third to ninth decades of life, with a mean of 58.4 years.^5^ Tubulocystic renal cell carcinoma is more commonly seen in the left kidney than the right and typically involves the renal cortex or corticomedullary junction.^5^ In our case, however, tubulocystic renal cell carcinoma occurred in a 21-year-old male in the right kidney and presented as a multifocal solid cystic mass.

Tubulocystic renal cell carcinomas are generally smaller than other variants such as clear cell, chromophobe, and papillary renal cell carcinoma; approximately 40% of the tumors are ≤2 cm.^1^ They are circumscribed, unencapsulated, and cortical in location and have been described as having a spongy, bubble-wrap, or Swiss cheese appearance because of the numerous variably sized cysts seen on the cut surface.^10^ In our case, almost the entire kidney was studded with variable-sized cysts except a small portion of normal renal parenchyma.

In 1955, tubulocystic renal cell carcinoma was thought to be a tumor of collecting duct origin and was sometimes confused with the collecting duct (Bellini duct) carcinoma.^9^ However, tubulocystic renal cell carcinoma and collecting duct carcinoma are not similar on immunohistochemical and ultrastructural studies. Tubulocystic renal cell carcinoma tumor cells are immunoreactive for proximal convoluted tubules markers (CD10 and P504S), distal tubules (CK19), and intercalated collecting duct cells (parvalbumin). Even on ultrastructure examination, tubulocystic renal cell carcinomas have features of microvilli with a brush border as seen in the proximal convoluted tubules but with short microvilli and cytoplasmic interdigitation, similar to intercalated cells of the collecting duct. Ultrastructure examination led Brennan and colleagues to hypothesize a renal tubule stem cells origin.^11^

Other cystic tumors of the kidney with a multiloculated gross appearance should be considered in the differential diagnosis: (1) multilocular cystic renal cell carcinoma, (2) cystic nephroma, (3) mixed epithelial and stromal tumors, (4) cystic oncocytoma, and (5) Xp11.2 translocation renal cell carcinoma. Variably sized cystic spaces are noted in multilocular cystic renal cell carcinoma and are lined by flattened to cuboidal clear cells, similar to those seen in clear cell renal cell carcinoma.^6^ Cystic nephroma is composed of multilocular cysts lined by flattened to attenuated epithelial cells; however, hobnailing is an infrequent feature.^12^ The mixed epithelial and stromal tumors have solid areas and broad septa, in contrast to the thin fibrous septa of tubulocystic renal cell carcinomas. The cystic oncocytomas usually show a tubulocystic pattern with numerous closely packed cystically dilated tubular structures, and, at least focally, a solid nest of oncocytic cells along with loose myxoid stroma.^5,13^ Xp11.2 translocation renal cell carcinoma with a tubulocystic pattern is an unusual tumor composed of large cysts with hyalinized stroma. Xp11.2 translocation renal cell carcinoma may have an ovarian-type stroma.^14^ Both tubulocystic renal cell carcinomas and cystic nephromas have cysts lined by hobnail cells, but the cells have a low nuclear grade in cystic nephroma, whereas tubulocystic renal cell carcinomas have a high-grade nuclear feature.

The clinical behavior of tubulocystic renal cell carcinomas is not certain. Four patients have developed metastatic disease per the literature.^1,15^ Of the 4 patients who developed metastases, Srigley and Delahunt^16^ reported that 2 patients developed distant metastases to the bone or liver. In the other study, 1 patient developed metastases to the pelvic lymph nodes with exclusive morphology of tubulocystic carcinoma in the metastatic nodes. Generally, these tumors are gradually progressive; however, a few of them behave aggressively.

A relationship has also been proposed between tubulocystic renal cell carcinoma and papillary renal cell carcinoma in view of similar molecular alterations noted in both the tumors (gains in chromosomes 7 and 17, loss of chromosome Y).^14,15^ Documentation of concomitant tubulocystic renal cell carcinoma and papillary renal cell carcinoma in the same kidney and foci of papillary renal cell carcinoma within tubulocystic renal cell carcinoma has supported this proposal.^16,17^ In our case, we also documented the presence of foci of papillary renal cell carcinoma.

The histomorphologic features of tubulocystic renal cell carcinoma are so distinctive that diagnosis can be established on morphology alone; however, the immunohistochemistry panel is required to determine the tumor's origin.^18^

The treatment of choice is radical nephrectomy; however, nephron-sparing surgery is an option for T1 stage tumors <7 cm. Because most tubulocystic renal cell carcinomas behave indolently, adjuvant therapy is generally not required. Only T3 and T4 stage tumors that have invaded the renal vein, inferior vena cava, and adrenal gland require adjuvant therapy. Close follow-up is recommended to look for any recurrences and metastases.^16^ Targeted therapy, including sunitinib and everolimus, is being explored for metastatic disease, but its role has not yet been well established.^19,20^

Stringent follow-up is required for cases in which focal clear cell, papillary, and sarcomatoid changes are observed. Only one case of tubulocystic carcinoma with sarcomatoid changes had been reported as of May 2021.^21^

## CONCLUSION

Cystic neoplasms of the kidney are uncommon and pose diagnostic challenges. Clinicians must consider the entire spectrum of differential diagnoses of all cystic entities of the kidney. Most cases of tubulocystic carcinoma are diagnosed on histomorphology alone, but immunohistochemistry is required to determine the origin of the tumor. Tubulocystic carcinomas behave in an indolent manner; however, occasional cases of recurrence and metastasis have been reported. In view of the few cases described in the literature, follow-up is recommended to precisely understand the biologic behavior of these tumors. No established guidelines are available for follow-up periods for tubulocystic carcinoma. Treatment options such as targeted therapy need to be explored.

## Notes

*This article meets the Accreditation Council for Graduate Medical Education and the American Board of Medical Specialties Maintenance of Certification competencies for Patient Care and Medical Knowledge.*
